# The Singularity of the Drosophila Male Germ Cell Centriole: The Asymmetric Distribution of Sas4 and Sas6

**DOI:** 10.3390/cells9010115

**Published:** 2020-01-03

**Authors:** Veronica Persico, Massimo Migliorini, Giuliano Callaini, Maria Giovanna Riparbelli

**Affiliations:** 1 Department of Life Sciences, University of Siena, Via Aldo Moro 2, 53100 Siena, Italy; persico@student.unisi.it (V.P.); migliorini@unisi.it (M.M.); riparbelli@unisi.it (M.G.R.); 2 Department of Medical Biotechnologies, University of Siena, Via Aldo Moro 2, 53100 Siena, Italy

**Keywords:** Drosophila, spermatogenesis, centriole asymmetry, Sas4, Sas6

## Abstract

Drosophila spermatocytes have giant centrioles that display unique properties. Both the parent centrioles maintain a distinct cartwheel and nucleate a cilium-like region that persists during the meiotic divisions and organizes a structured sperm axoneme. Moreover, the parent centrioles are morphologically undistinguishable, unlike vertebrate cells in which mother and daughter centrioles have distinct structural features. However, our immunofluorescence analysis of the parent centrioles in mature primary spermatocytes revealed an asymmetric accumulation of the typical Sas4 and Sas6 proteins. Notably, the fluorescence intensity of Sas4 and Sas6 at the daughter centrioles is greater than the intensity found at the mother ones. In contrast, the centrioles of wing imaginal disc cells display an opposite condition in which the loading of Sas4 and Sas6 at the mother centrioles is greater. These data underlie a subtle asymmetry among the parent centrioles and point to a cell type diversity of the localization of the Sas4 and Sas6 proteins.

## 1. Introduction

Centrioles are widely conserved barrel shaped organelles present in almost all the organisms. They are involved in the organization of the cytoplasmic microtubule network in interphase and during the cell division by recruiting the molecules need to their nucleation. Moreover, the centrioles are required to assembly cilia and flagella [1]. Due to the importance of the microtubule scaffold in several aspects of the cell life, any defect in centriole structure can lead to cell abnormalities that in humans may result in important diseases [2,3,4,5,6].

Each sister cell inherits at the end of division a pair of different aged parent centrioles that in vertebrate tissues are characterized by distinctive morphological traits [7]. The older mother centriole carries distal and subdistal appendages. The distal appendages mediate the docking to the cell membrane during primary cilia assembly [8] and immunological synapse formation [9]. The subdistal appendages play main roles in the recruitment of the pericentriolar material and the molecules need to assembly the cytoplasmic microtubule network in interphase and during cell division [10]. The daughter centriole acquires such specializations one cell cycle later when it will be in turn mother. This structural asymmetry is strengthened by a distinct molecular composition of the parent centrioles [11]. Some proteins, such as ninein [12], Odf2/cenexin [13], centriolin [14], and Cep164 [15] are found only on the mother centriole while others like centrobin [16], Neur14 [17], and Cep120 [18] are specific of the daughter. Remarkably, the centriolar asymmetry is correlated in vertebrate tissues to different functional aspects, such as the specific recruitment of γ-tubulin complexes [19], the nucleation of primary cilia [20], and the unequal fate of the sister cells following the asymmetric division of the radial glial cells [21,22].

In Drosophila the parent centrioles differently influence the microtubule nucleating properties of the centrosome, thus defining the orientation of the mitotic spindle that ensures stemness or differentiation in male germ cells [23] and in larval neuroblasts [24]. Indeed, the daughter centriole recruits more pericentriolar material during the asymmetric division of larval neuroblasts [25], whereas the mother centrosome organizes the larger aster of microtubules in male germ stem cells [26]. Therefore, the different age of the parent centrioles impacts in Drosophila also with the activity of the centrosome and has important outcomes on the binary decision fate of the sister cells. However, although mother and daughter centrioles may be endowed with different functional aspects, they lack a distinct structural dimorphism and unlike mammalian cells there are no visible structures associated with the external microtubule wall [27]. The Drosophila cells do not assemble true primary cilia and thus distal appendages could be redundant. Remarkably, all the Drosophila centrioles, apart from the ciliated sensory neuron centrioles [28], maintain a distinct cartwheel. Conversely, in vertebrate cells, the cartwheel is a scaffolding structure that is transiently present in the basal region of the daughter centrioles and is soon lost during the centriole-to-centrosome conversion process [29].

However, despite the parent centrioles in Drosophila tissues are morphologically undistinguishable some differences in their molecular composition have been occasionally reported. The scaffolding centrosomal protein Dplp is preferentially recruited to the mother centrioles of somatic cells [30,31], whereas centrobin specifically accumulates to the daughter centrioles in larval neuroblasts [32], sensory neurons [33], and ommatidial cells [34]. Remarkably, centrobin discriminates the parent centrioles in female germ stem cells [35] and during the spermatogonial divisions [36], but it is found at the proximal region of both the parent centrioles in mature primary spermatocytes [36]. Moreover, the accumulation of Dplp to the parent centrioles of primary spermatocytes does not reveal appreciable differences [37] and both the parents are able to organize cilium-like structures [38], a function only reserved to the mother centrioles in vertebrate cells. These findings point to the loss of identity among mother and daughter centrioles during male meiosis. However, a transient accumulation of the conserved centriole duplication factor Ana2 to the daughter centrioles has been observed in primary spermatocytes [39] and depletion of the kinesin-like protein *Klp10A*, a key regulator of centriole length in Drosophila [40], results in shorter daughter centrioles during spermatogenesis [41,42]. Thus, it is unclear if the different age of the parent centrioles in primary spermatocytes could be correlated with an intrinsic asymmetry. To verify this possibility, we examined the localization of Sas4 and Sas6, two conserved proteins mainly involved in centriole assembly and elongation. Moreover, we also look at the distribution of γ-tubulin during male meiosis to eventually underline different functional aspects of the parent centrioles.

Although, there are no appreciable differences among the parent centrioles in the distribution of γ-tubulin to the parent centrioles during male meiosis, we noticed that both Sas4 and Sas6 are enriched at the basal regions of the daughter centrioles, pointing to a new underscored asymmetry during Drosophila spermatogenesis. Conversely, Sas4 and Sas6 are enriched to the mother centrioles in somatic cells supporting a subtle molecular diversity among germline and somatic cell centrioles.

## 2. Materials and Methods

### 2.1. Drosophila Strains

The stocks containing the Unc-GFP and the Sas6-GFP transgenes were described previously [43,44]. Flies were raised on standard Drosophila medium at 24 °C.

### 2.2. Reagents

We used the following antibodies, mouse anti-γ-tubulin-GTU88 (1:100), mouse anti-acetylated tubulin (1:100; Sigma-Aldrich, St. Louis, MO, USA); rabbit anti-Spd2 (1:500; [45]); chicken anti-Dplp (1:1500; [45]); mouse anti-Sas4 (1:200; [46]). The secondary antibodies used were Alexa Fluor-488- and Alexa Fluor-555-conjugated anti-mouse-IgG, anti-rabbit-IgG, anti-chicken-IgG (1:800; Invitrogen, Waltham MA, USA). Dimethyl sulfoxide (DMSO) and Shields and Sang M3 Insect Medium were purchased from Sigma-Aldrich. MLN8054 and BI2536 were obtained from Selleck and were dissolved in DMSO at a stock concentration of 1000 mM and stored frozen at 20 °C. The stock solution was diluted to the desired concentration in the culture medium prior to incubation with testes.

### 2.3. Culture and Drug Treatment Experiments

Testes were dissected from mid-aged pupae in M3 medium. To inhibit Aurora A and Polo kinases, testes were incubated 24 h in M3 medium containing 1 mM MLN8054 or 100 nM BI2563 into a 24-well plate at 24 °C. Incubation of testes in M3 medium containing DMSO but lacking MLN8054 or BI2563 had no effect on the recruitment of the pericentriolar material during meiosis.

### 2.4. Immunofluorescence Staining

Dissected testes were placed in a small drop of 5% glycerol in PBS on a glass slide and squashed under a small cover glass and frozen in liquid nitrogen. After removal of the coverslip, the samples were immersed in cold methanol for 10 min. For localization of centrosomal components, testes were washed for 15 min in PBS and incubated for 1 h in PBS containing 0.1% bovine serum albumin (PBS-BSA) to block nonspecific staining. Then, the samples were incubated in the specific antibodies overnight at 4 °C. After washing in PBS–BSA, the samples were incubated for 1 h at room temperature with the appropriate secondary antibodies. In all cases, DNA was visualized with incubation of 3–4 min in Hoechst. Testes were mounted in small drops of 90% glycerol in PBS.

### 2.5. Transmission Electron Microscopy

Testes from mid-aged pupae were pre-fixed in 2.5% glutaraldehyde in phosphate buffered saline (PBS) overnight at 4 °C. Samples were rinsed in PBS and subsequently post-fixed in 1% osmium tetroxide in PBS for 1 h at 4 °C. After rinsing in PBS, the material was dehydrated through a graded series of ethanol, infiltrated with a mixture of Epon–Araldite resin and polymerized at 60 °C for 48 h. Serial ultrathin sections were cut with a Reichert ultramicrotome equipped with a diamond knife, collected with formvar-coated copper grids, and stained with uranyl acetate and lead citrate. TEM preparations were observed with a FEI Tecnai G2 Spirit transmission electron microscope operating at 100 kV and equipped with a Morada CCD camera (Olympus, Shinjuku, Tokyo, Japan).

### 2.6. Image Acquisition and Data Analysis

Images were taken by using an Axio Imager Z1 (Carl Zeiss, Jena, Germany) microscope equipped with an AxioCam HR cooled charge-coupled camera (Carl Zeiss). Grayscale digital images were collected separately and then pseudocolored and merged using Adobe Photoshop 7.0 software (Adobe Systems, San Jose, CA, USA). The analysis of Sas6 and Sas4 fluorescence signal intensity was performed only on centriole pairs in which it was possible to unambiguously recognize the single parents.

As it was difficult to have comparable data of the fluorescence intensity of Sas4 and Sas6 from different preparations, due to the background and the variable antibody reactivity, we measured the signal intensity at the mother and the daughter centrioles within each pair and calculated the mutual ratio value. The fluorescence intensity was quantified by the image processing software Image J (National Institutes of Health, Bethesda, MD, USAusing plugins that allowed us to delineate small ring areas to precisely measure the fluorescent spots. All data analyses utilized the Excel (Microsoft Italy, Office 2016, Milano, Italy) software. Mean ± s.d. is reported for all distributions of data.

## 3. Results

### 3.1. The Proximal Region of the Giant Drosophila Spermatocyte Centriole Is the Main Site for Microtubule Nucleation

**Figure 1:**
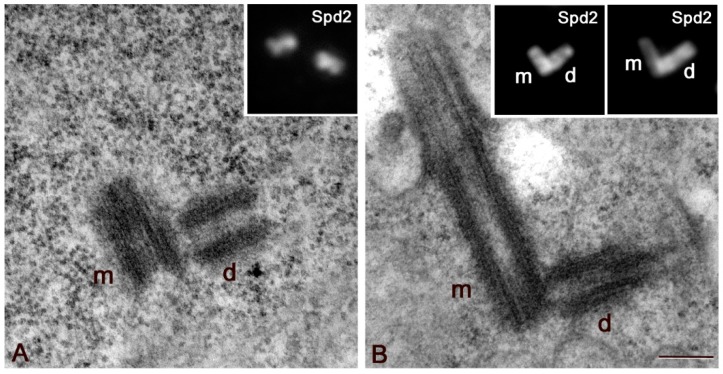
Centriole growth in primary spermatocytes. Ultrastructural details of centrioles in early (**A**) and mature (**B**) primary spermatocytes showing that the daughter centriole (d) lies orthogonal to the proximal end of its mother (m). This orthogonal disposition is not obvious in immunofluorescence analysis of early spermatocytes ((**A**), inset), but becomes apparent when the centrioles elongate ((**B**), insets). m and d, mother and daughter centrioles, respectively. Scale bar: (**A**,**B**), 200 nm; insets, 1 μm.

The daughter centrioles are assembled in young Drosophila primary spermatocytes at right angles to their mothers (Figure 1A). As prophase progressed, both the parent centrioles elongate and the orthogonal orientation of the daughter to the proximal region of the mother is well evident (Figure 1B). Although the orthogonal disposition is always clear in ultrastructural observations, the reciprocal orientation of the centrioles is not obvious in immunofluorescence analysis of young primary spermatocytes when the centrioles are small (Figure 1A, inset). However, in mature primary spermatocytes, the orthogonal disposition is evident (Figure 1B, insets) and the centriole sitting perpendicularly to the basis of the other parent has be recognized as the daughter [39,47]. This orthogonal disposition persists until the onset of the first anaphase when the centrioles disengage and lose their reciprocal orientation, making it impossible to distinguish the single parents. The large centrioles of the Drosophila spermatocytes that reach a length of about 1 μm at the onset of the first prometaphase, may represent a suitable model in which to decipher the spatial localization of the proteins involved in microtubule nucleation and centriole organization.

**Figure 2:**
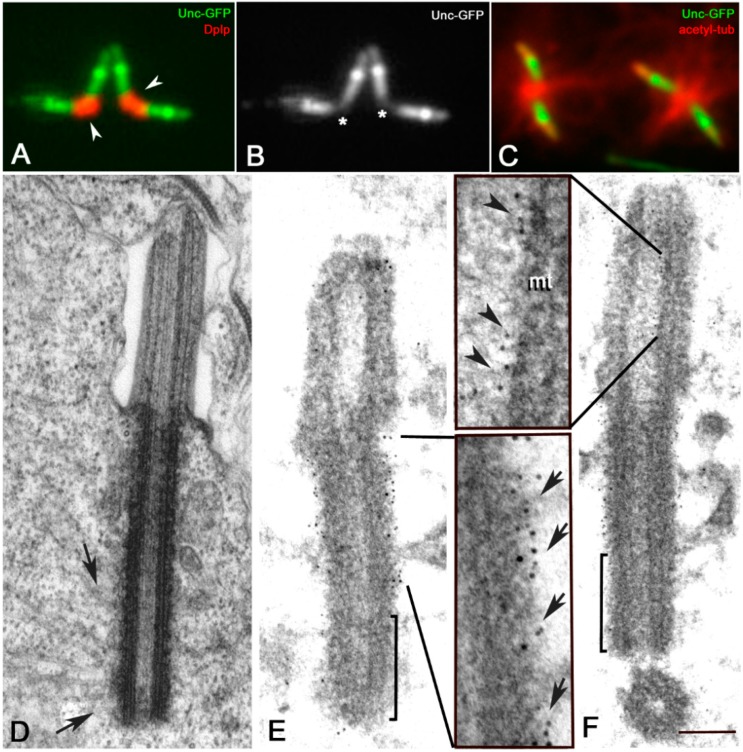
The proximal region of the centrioles is the main site for microtubule nucleation in mature primary spermatocytes. (**A**) Dplp is restricted to the proximal region of the centrioles (arrowheads). (**B**) Unc-GFP is localized in three distinct domains along the centrioles of mature primary spermatocytes but it is absent from their basal region where Dplp accumulates (asterisks). (**C**) Microtubules are organized in aster-like fashion from the basis of the parent centrioles (arrows). (**D**) Longitudinal section of a centriole/CLR from a mature primary spermatocyte: most of the microtubules originate from the proximal region of the centriole (arrows). (**E**,**F**) Ultrastructural localization of the Unc protein on centrioles of primary spermatocytes: the gold particles are enriched on the outer middle and distal regions of the centrioles (arrows), but lack from the proximal region (brackets); gold particles (arrowheads) are found inside the CLR adjacent to the microtubules (mt) of the ciliary axoneme. Scale bar: (**A**–**C**), 1 μm; (**D**–**F**), 200 nm; insets 50 nm.

The proximal region of the parent centrioles is specifically recognized by an antibody against the PACT residue of the Drosophila pericentrin-like protein (Dplp) [37,48,49] (Figure 2A,B). As this protein is involved in scaffolding the pericentriolar material [31,50], the proximal region of the meiotic centrioles may represent the main site for microtubule nucleation.

Immunofluorescence analysis of mature primary spermatocytes with an antibody against acetylated tubulin showed, indeed, that most of the microtubules were nucleated from the proximal region of the parent centrioles (Figure 2C). This is also confirmed by ultrastructural analysis in which we find several microtubules associated with the outer wall of the basal region of the centrioles (Figure 2D).

We wondered, therefore, whether the whole wall or only a restricted region of the elongated centrioles can nucleate the microtubules. As this process is strictly dependent by γ-tubulin, we examined the distribution of this protein on centrioles of primary spermatocytes (*n* = 187) from testes of transgenic flies expressing a Unc-GFP construct which allows to recognize distinct centriole regions. As previously reported the Unc-GFP signal is localized, indeed, in three distinct domains along the centriole/cilium like regions of the mature Drosophila spermatocytes but is absent from the proximal region [38] (Figure 2B). Immunoelectron microscopy analysis of primary spermatocytes expressing Unc-GFP (*n* = 73) confirmed this observation. The gold particles that precisely delineate the localization of Unc-GFP signal were, indeed, associated to the outer wall of the middle and the distal regions of the centrioles, but lack within the proximal region (Figure 2E,F). Although, the conventional immunofluorescence showed a rather uniform signal along the centriole/CLR complex, the gold particles were found outside the centriole wall (Figure 2E) and inside the CLR lumen (Figure 2F).

**Figure 3:**
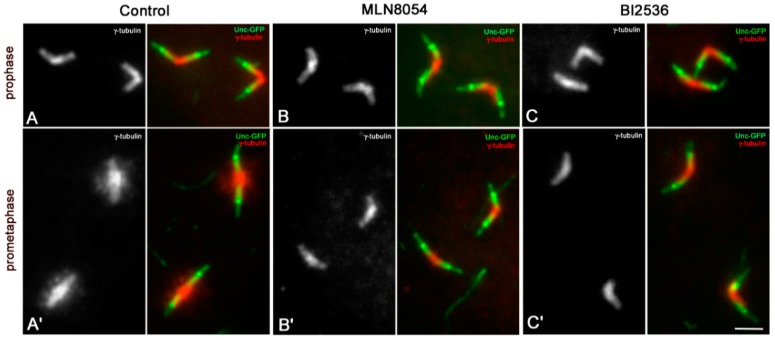
Aurora A and Polo/Plk1 kinase inhibition does not affect the accumulation of γ-tubulin in prophase spermatocytes. Double labelling with Unc-GFP shows that γ-tubulin is diffused along the whole centriole wall, with a distinct accumulation to the proximal regions of both the parent centrioles (**A**). The accumulation of γ-tubulin does not change following incubation with MLN8054 (**B**) and BI2536 (**C**), small molecules that inactivate the Aurora A and Polo/Plk1 kinases, respectively At the onset of the meiotic division the amount of γ-tubulin increased in control spermatocytes (**A’**) but remains at the levels of the previous prophase after depletion of Aurora A (**B’**) and Polo/Plk1 (**C’**). Scale bar: 1 μm.

γ-tubulin was found along the whole centrioles in mature primary spermatocytes with a distinct accumulation to their proximal regions (Figure 3A). The amount of this protein increased during prometaphase, when the meiotic spindle starts to be assembled (Figure 3A’). However, γ-tubulin was still enriched at the basal regions of the parent centrioles in agreement with previous analysis of γ-tubulin recruitment during male spermatogenesis [37,51,52]. γ-tubulin accumulation to mother and daughter centrioles did not show appreciable differences in all the spermatocytes scored (*n* = 273).

As Aurora A and Polo/Plk1 kinases are involved in the recruitment of the pericentriolar material during centrosome maturation [53], we ask whether their inhibition during Drosophila male meiosis could reveal different basal levels of γ-tubulin among the parent centrioles. Treatment of male germ cells with MLN8054 and BI2536, small molecule kinase inhibitors of Aurora A [54], and Polo/Plk1 [55], respectively, had significant effects on the recruitment of γ-tubulin at the onset of cell division. We find that the distribution of γ-tubulin to the centrioles of mature prophase spermatocytes was unperturbed by the inhibition of the Aurora A (Figure 3B; *n* = 94) and Polo/Plk1 (Figure 3C; *n* = 76) kinases, but its accumulation at prometaphase was dramatically reduced following MLN8054 (Figure 3B’; *n* = 85) or BI2536 (Figure 3C’; *n* = 91) treatment. The accumulation of γ-tubulin in treated prometaphase spermatocytes was very similar to that found in treated and untreated mature primary prophase spermatocytes and did not differ between the parent centrioles. This suggests that Aurora A and Polo/Plk1 are key to γ-tubulin recruitment during meiotic division, but dispensable to maintain the basal pool of this protein at the centriole before the onset of the spindle assembly.

### 3.2. Sas6 and Sas4 Are Enriched to the Proximal Region of the Daughter Centrioles during Male Meiosis

**Figure 4:**
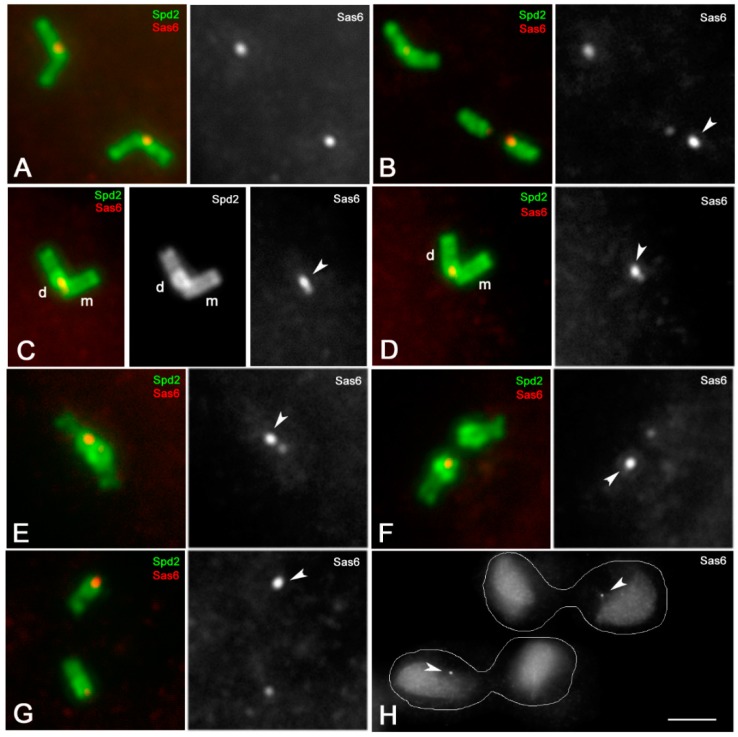
Localization of Sas6 during meiotic progression. Centrioles expressing Sas6-GFP (red) are counterstained with an antibody against Spd2 (green). The brighter Sas6-GFP spots (arrowheads) are associated with the daughter centrioles. (**A**–**D**) Mature primary spermatocytes. The centrioles in panel (**C**) are a representative pair in which the daughter (d) is orthogonal to the basal region of the mother (m). (**E**) Metaphase I. (**F**) Anaphase I. (**G**) Telophase I. (**H**) Only one sister cell of the secondary telophase spermatocytes inherits the brighter Sas6-GFP dot (arrowheads). m and d, are mother and daughter centrioles, respectively. Scale bar: (**A**–**G**); 2 μm; (**H**), 10 μm.

The above findings suggest a specific function of the proximal region of the spermatocyte centrioles, and led us to verify whether this region displays peculiar features. We then analyzed the localization of Sas6, the master protein for cartwheel assembly. Since both mother and daughter centrioles of the Drosophila spermatocytes have a distinct cartwheel, Sas6 would be localized to the basal regions of both the centrioles. In testes expressing a Sas6-GFP transgene, and counterstained with an antibody against Spd2 to outline the centriole wall, we find, indeed, a distinct signal that appeared as a large dot associated with the adjacent bases of the centriole pairs (Figure 4A). However, when one of the centrioles occasional moved away in primary spermatocytes (*n* = 21) due to the squashing preparation, the amount of the endogenous Sas6 protein to the parents roughly differed (Figure 4B). This is notably in mature primary spermatocytes in which one of the centrioles of each pair displayed one brighter Sas6-GFP spot. This observation led us to investigate which of the parent centriole accumulated more Sas6 protein. As the Drosophila centrioles lack a distinct structural dimorphism, we addressed the possibility to recognize the parents by their relative spatial orientation: the daughter centrioles are always orthogonal to the basal region of their mothers. We thus performed a careful analysis of mature primary spermatocytes in search of centriole pairs where the parents can be recognized by their relative position. We noticed that in all the centriole pairs examined in which the parents were easily distinguishable, the brighter Sas6 dot was always associated with the daughter centriole (Figure 4C,D). The mean value of the Sas6 fluorescence intensity ratio between the daughter and the mother centrioles measured in 129 centriole pairs ranged from a minimum of 1.27 and a maximum of 2.21 (mean value 1.72 ± 0.24).

The asymmetric accumulation of Sas6 to the parent centrioles persisted in metaphase spermatocytes (*n* = 87 spermatocytes) (Figure 4E) and became more evident as the centrioles disengaged at the onset of first anaphase (*n* = 64 spermatocytes) (Figure 4F). During telophase of the first meiosis (*n* = 47 spermatocytes), each spindle pole contains two separated centrioles, but only one of them showed a higher Sas6-GFP signal (Figure 4G). The centrioles do not duplicate during the second prophase and each spindle pole inherits one centriole after the second telophase (*n* = 88 spermatocytes). Only one of the two sister cells retained the brighter Sas6-GFP signal (Figure 4H). Consequently, about half of the young spermatids scored (101; *n* = 211) showed a brighter Sas6-GFP dot (not shown).

**Figure 5:**
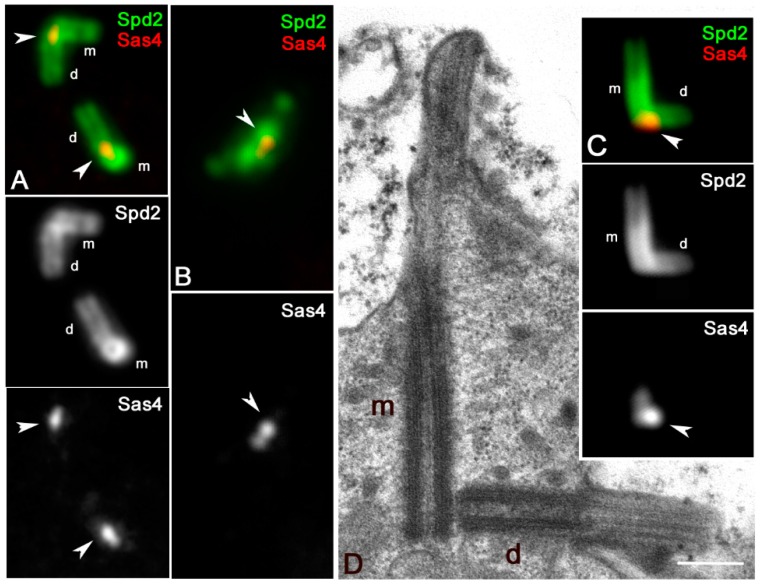
Localization of Sas4 in mature primary spermatocytes. (**A**) Detail of the centrioles in a mature primary spermatocyte in which it is possible to recognize mothers (m) and daughters (d) by their reciprocal orientation: Sas4 is more concentrated at the proximal ends of the daughter centrioles (arrowheads). (**B**) Detail of one centriole pair during the first metaphase showing that the asymmetric accumulation of Sas4 persists (arrow), but the parent centrioles are no longer distinguishable. (**C**) Longitudinal sections of the parent centrioles in Klp10A mutant primary prophase spermatocytes showing their asymmetric elongation. (**D**) The Sas4 brighter dot is associated with the shorter daughter centriole (arrowhead). m and d, are mother and daughter centrioles, respectively. Scale bar: (**A**–**C**), 1 μm; (**D**), 200 nm.

We then look at the localization of Sas4, the master protein for centriole elongation that in mature Drosophila spermatocytes is restricted to the basal region of both the parent centrioles close to the cartwheel [37]. By double-labeling primary spermatocytes with antibodies against Spd2, to delineate the centriole wall, we find one small dot of Sas4 at the basis of each centriole (Figure 5A). However, we noticed that when the parent centrioles of the primary spermatocytes were easily recognizable within each pair by their reciprocal orientation (*n* = 89 spermatocytes) the Sas4 signal associated with the mother centriole is weaker than that associated with the daughter one (Figure 5A). The asymmetric loading of Sas4 became more evident at metaphase (Figure 5B) even if the parents were no longer distinguishable. The mean value of the ratio of the Sas4 fluorescence intensity between the daughter and the mother centrioles measured in 79 centriole pairs from first prophase spermatocytes ranged from a minimum of 1.15 and a maximum of 1.75 (mean value 1.37 ± 0.14).

To confirm this asymmetric enrichment, we look at the Sas4 localization in mature spermatocytes of *Klp10A* flies, a kinesin-like protein involved in centriole length control. The daughter centrioles are usually shorter in *Klp10A* mutant spermatocytes (Figure 5C,D), thus making easy to identify the parents in each centriole pair. All the mutant primary spermatocytes scored in which the parents were unambiguously distinguished (*n* = 63) showed a brighter Sas4 dot that was always associated with the shorter centriole (Figure 5C).

### 3.3. Sas6 and Sas4 Are Enriched to the Mother Centrioles in Somatic Cells

**Figure 6:**
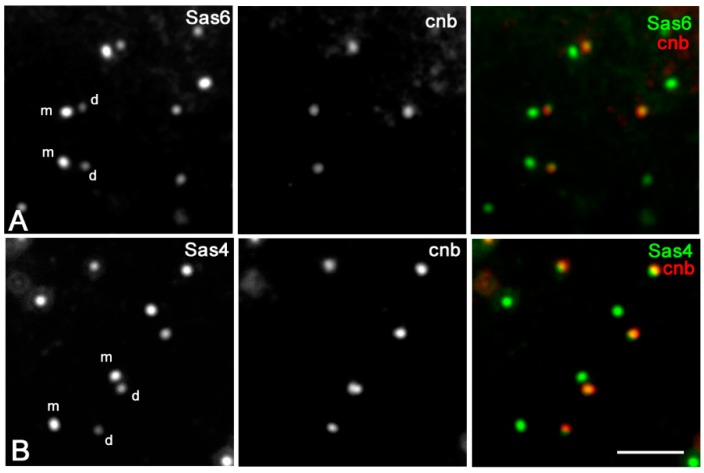
Sas6 and Sas4 loading in third larval imaginal wing cells. Double labeling with an anti-centrobin antibody (cnb, red) that specifically recognizes the daughter centrioles, shows that the immunofluorescence intensities of both Sas6-GFP ((**A**), green) and Sas4 ((**B**), green) at the mother centrioles are greater than the intensity to the daughter centrioles. m and d, are mother and daughter centrioles, respectively. Scale bar: 1 μm.

We then asked whether the centrioles of the somatic cells showed the same asymmetric accumulation of Sas6 and Sas4 observed during male meiosis. For this purpose, we examined the distribution of these proteins in the wing imaginal discs from third-instar larvae. Mother and daughter centrioles are very small, and their respective orientation cannot be resolved by the conventional immunofluorescence microscopy. However, parental centrioles in somatic tissues can be easily discriminate among them, as the daughters selectively express the protein centrobin that it is absent from the older mother centriole. Surprisingly, and in contrast to male gametogenesis, we find that the immunofluorescence intensity of Sas6 (Figure 6A) and Sas4 (Figure 6B) at the mother centrioles was greater than the intensity at the daughter centrioles. The mean value of the Sas6 fluorescence intensity ratio between the mother and the daughter centrioles scored in 141 centriole pairs from five different wing discs ranged from a minimum of 1.27 and a maximum of 2.33 (mean value 1.61 ± 0.33). The mean value of the ratio of the Sas4 fluorescence intensity scored in 97 centriole pairs from 3 different wing discs ranged from a minimum of 1.39 and a maximum of 1.93 (mean value 1.67 ± 0.20).

## 4. Discussion

The extension of the Dplp signal does not change significantly during the process of centriole elongation, and it mirrors the extension of the cartwheel that is present in both the parent centrioles and remains roughly constant during meiotic progression [37]. The accumulation of γ-tubulin in correspondence of the Dplp signal suggests that the cartwheel region may represent a preferred site for microtubule nucleation during meiosis, in addition to its role in centriole assembly and biogenesis. It has been showed, indeed, that Dplp plays a role in the assembly of a discrete electron-dense material that emanate from the cartwheel spokes of the prophase spermatocyte centrioles to organize the cytoplasmic microtubule network [50]. Therefore, the centrioles of the mature primary spermatocytes consist of two distinct regions: the proximal region where Sas4, Sas6 and Dplp are concentrated and the elongating mid-distal region continuous with the ciliary axoneme. These observations suggest that the true spermatocyte centriole, from which most of the cytoplasmic microtubules are nucleated, could be restricted to the region of the cartwheel, whereas the region in which Unc-GFP is expressed might represent a potential modified ciliary extension.

Both the parent centrioles of the Drosophila primary spermatocytes have a distinct cartwheel. Therefore, it is surprising that the daughter centriole recruits more Sas6. However, the different accumulation of this protein is not due to a reduction of the cartwheel of the mother centriole. Our preliminary ultrastructural analysis of primary spermatocytes did not, indeed, revealed appreciable differences between the cartwheels of the parent centrioles (not shown). Otherwise, the asymmetric accumulation of Sas6 might reflect the specific interactions with different centriolar proteins. Remarkably, the conserved centriole duplication factor Ana2 that interacts with Sas-6 and plays a main role in its recruitment [56,57,58,59,60,61] is also more concentrated to the daughter centrioles in primary spermatocytes [39]. However, whereas Sas-6 is restricted to the proximal region of the centriole, Ana2 is localized along the whole centriole length [39]. Moreover, Ana2 is lost by the centrioles at the onset of meiosis whereas the asymmetric concentration of Sas6 does not change over time.

Sas-6 is recruited in cycling vertebrate cells by the forming daughter centrioles that at the onset of mitosis have a distinct cartwheel. However, the cartwheel is lost when the centriole undergoes the centriole-to-centrosome conversion at the end of mitosis [39], and the Sas6 signal is no longer visible. In Drosophila the mother centriole also displays a cartwheel and accumulates Sas6. Due to the basal amount of Sas6 to the mother it is possible that the formation of a new daughter needs more protein to avoid uncontrolled centriole duplication. Thus, the asymmetric distribution of Sas6 might be correlated to the unusual dynamics of the centrioles during male meiosis in Drosophila.

Usually the animal cells maintain two centrosomes over generations by synchronizing the duplication of their centrioles with the DNA cycle [53]. However, this general rule is turned out during male meiosis of most organisms in which the centrioles duplicate between meiosis I and II in the absence of DNA replication and the differentiating spermatids inherit two distinct centrioles that may undergo morphological and structural modifications during the following spermiogenesis [62]. Spermatogenesis in Drosophila represents a notable exception in that centrioles duplicate only once at the beginning of the first meiotic prophase [63]. Thus, only one parent centriole is inherited at the end of meiosis, explaining why only half of the spermatids display a more fluorescent Sas6 spot.

The accumulation of Sas6 at the time of duplication could be maintained through meiotic progression by the daughter centrioles unless new duplication events occur. All the parent centrioles resume their competence to duplicate at the onset of spermatid differentiation when a short procentriole assemble at their proximal end [64,65].

The Sas4 protein that is specifically involved in centriole length control by directing the assembly of the centriole microtubule wall [66,67,68,69] also binds Ana2 [70,71,72] to help its activation [73]. Therefore, the concentration of Sas6 at the parent centrioles in Drosophila spermatocytes could reflect the loading of Ana2 that in turn depends on the Sas4 recruitment. Accordingly, we find a higher accumulation of Sas4 at the daughter centrioles in primary spermatocytes. Unlikely, the asymmetric loading of Sas4 and Sas6 could reflect different functional aspects of the parent centrioles. Both mother and daughter centrioles express centrobin [36], recruit the same amount of γ-tubulin at their base, and organize in primary spermatocytes the cilium-like regions that convert in true axonemes at the onset of spermatid differentiation [74].

Conversely, the mother centrioles of wing imaginal disc cells recruit more Sas4 and Sas6 than their daughters. Although, the significance and the mechanisms responsible for this asymmetric recruitment are unclear, the divergent condition observed in germ cells and imaginal discs could reflect an intrinsic variability between the centrioles of various tissues. Somatic cell centrioles that undergo repeated duplication cycles accumulates more Sas4 and Sas6 to the mother centrioles, whereas these proteins are enriched to the daughter centrioles in spermatocyte centrioles that do not undergo further duplication during the meiotic divisions.
